# Edge states jointly determined by eigenvalue and eigenstate winding

**DOI:** 10.1038/s41377-025-02038-y

**Published:** 2025-09-30

**Authors:** Jinbing Hu, Yixin Sha, Yi Yang

**Affiliations:** 1 School of Optical-Electrical and Computer Engineering, University of Shanghai for Science and Technology, Shanghai, China; 2 Department of Physics and HK Institute of Quantum Science and Technology, The University of Hong Kong, Pokfulam, Hong Kong, China

**Keywords:** Optical physics, Optical physics

## Abstract

A photonic synthetic angular-momentum lattice realizes non-Hermitian topological edge modes that are jointly determined by the eigenstate and eigenenergy winding numbers.

In Hermitian systems, single-particle topological invariants are quantized to integers, like the winding number and Chern number^1^. Nevertheless, non-Hermitian systems^2–4^, which incorporate energy exchange with external environments, can exhibit fractional topological invariants like half-integers. Theoretical studies have predicted that in one-dimensional non-Hermitian lattices, two distinct winding numbers can be defined: one based on eigenvectors (denoted as *w*, akin to the Zak phase)^3,5,6^ and another based on complex energy (denoted as *v*)^7^. The combinations of these two types of winding numbers govern the presence of edge states under open-boundary conditions. For example, *v* = 0 and *w* = 0 yielding no edge states, *v* = 0 and *w* = 1 producing two edge states at both ends (Fig. 1a), and |*v* | = 1 with *w* = 1*/*2 resulting in a single edge state in a semi-infinite chain (Fig. 1b), highlighting their unique behavior^4,5^. So far, the experimental demonstration of these predictions has been lacking.

**Fig. 1:**
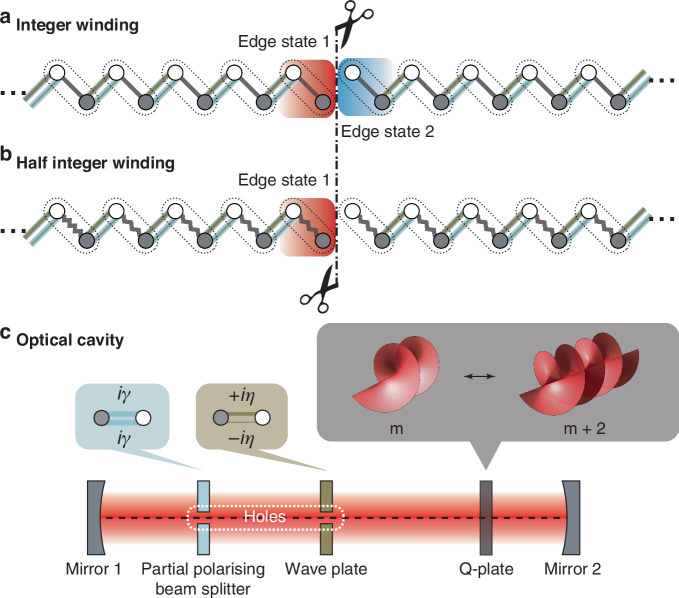
Non-Hermitian lattice model via the synthetic orbital angular momentum dimension and bulk-boundary correspondence. **a** The topological edge states at both ends of the non-Hermitian OAM lattice chain when *v* = 0 and *w* = 1. **b** A single edge state at the single end of a semi-infinite OAM lattice chain when *v* = −1 and *w* = 1*/*2. **c** Schematic figure of a degenerate optical cavity composed of a Q-plate, a wave plate, and a partially polarized beam splitter. The Q-plate generates intracell hoppings among distinct lattice sites by partially transferring spin to orbital (i.e., *m*) of resonant modes. The wave plate induces intercell hoppings among OAM modes, and the partially polarized beam splitter introduces non-Hermiticity. By drilling a proper hole at the center of both the wave plate and the beam splitter, two semi-infinite lattice chains are created, in which a single edge state jointly determined by *v* and *w* (i.e., panel b) is observable

A recent study by Yang et al.^8^, realized non-Hermitian edge modes jointly controlled by the eigenstate and eigenvalue winding numbers. The team successfully created a one-dimensional non-Hermitian lattice model using synthetic dimensions^9,10^ constructed from orbital angular momenta (OAMs) in an optical cavity^11,12^ (Fig. 1c). By leveraging the OAM of light as lattice sites, the researchers introduced non-Hermiticity via a partially polarized beam splitter and provided the experimental evidence of this half-integer non-Hermitian topological invariant *w* = 1*/*2. Unlike prior experiments observing half-integer winding numbers in the vicinity of exceptional points, the half-integer valued eigenstate winding number observed in this study is defined across the full Brillouin zone. Importantly, a semi-infinite chain was realized, verifying a single edge state of the half-integer eigenstate winding number.

To create a domain wall, the team partitioned the infinite OAM chain into two semi-infinite chains by strategically drilling a proper hole in the wave plate and partially polarized beam splitter, blocking the propagation of higher-order OAM modes (Fig. 1c). This setup effectively mimicked the open-boundary condition of a semi-infinite lattice (Fig. 1b)^13^, enabling the observation of edge-state characteristics of such systems. The authors performed polarization-resolved transmission measurements to map out both eigenenergy and eigenstate windings. The experiment directly confirmed the theoretical prediction that for *w* = 1*/*2 and |*v*| = 1, a single edge state emerges at the end of the semi-infinite chain, with its position determined by the sign of *v*. This work represents the experimental validation of the correspondence between half-integer winding numbers and edge states in semi-infinite non-Hermitian chains, bridging a critical gap between theories and experiments.

The reported measurement incorporates both spectral and transport characteristics. Such high controllability in synthetic non-Hermitian systems may find potential applications in a broader context. The flexibility of the synthetic dimension framework, particularly using OAM modes, offers a versatile platform for exploring photonic topological phenomena, such as edge modes of semi-infinite domain wall configurations of non-Hermitian quantum walks^14^ and the interplay between non-Hermiticity and quantum entanglement^15,16^. Moreover, the polarization-resolved and quasi-momentum-resolved (relating to the azimuthal coordinate of light in this case) measurement performed here could be particularly useful for creating non-Abelian topology^17^ based on the OAM of light.
